# Systemic Capillary Leak Syndrome Induced by Influenza Type A Infection: A Case Report

**DOI:** 10.7759/cureus.34213

**Published:** 2023-01-25

**Authors:** Masafumi Fukuda, Masakazu Nabeta, Nobuhisa Hirayu, Mikinori Kannae, Osamu Takasu

**Affiliations:** 1 Intensive Care Unit, Advanced Emergency and Critical Care Center, Kurume University Hospital, Kurume, JPN

**Keywords:** acute kidney injury, renal replacement therapy (rrt), influenza virus type a, rhabdomyolysis, systemic capillary leak syndrome

## Abstract

Rhabdomyolysis accompanying influenza virus infection is a notable extrapulmonary complication. We experienced a case of influenza type A followed by rhabdomyolysis and systemic capillary leak syndrome (SCLS). A 57-year-old man with no significant past medical history was diagnosed as having influenza type A six hours after fever onset, and treatment with oseltamivir was started. Shock, rhabdomyolysis, and acute kidney injury (AKI) progressed rapidly. At 53 hours after starting the oral treatment, intensive care was initiated, including ventilation management. In the acute phase, a large-dose replacement was given for the SCLS and continuous renal replacement therapy for AKI; both eventually healed without sequelae.

## Introduction

Influenza has a wide spectrum of clinical presentations, from mild upper respiratory tract inflammation to acute respiratory distress syndrome. Influenza causes extrapulmonary complications like encephalitis, myocarditis, pericarditis, and rhabdomyolysis [1]. We herein report a rare case of significant muscle damage to the trunk and rhabdomyolysis after influenza type A infection that was complicated by systemic capillary leak syndrome (SCLS) [2].

## Case presentation

A 57-year-old male with no significant past medical history presented to the hospital with the chief complaint of fever for six hours. The patient was diagnosed with influenza type A and was notably unvaccinated for influenza. He was started on oseltamivir and discharged from the outpatient department to follow up on an outpatient basis. Symptoms progressed to muscle aches and malaise so he returned to the hospital at 18 hours and was admitted for further management. His blood pressure on admission was 78/53 mmHg, respiratory rate was 30 breaths/min, peripheral capillary oxygen saturation was 90%, and his body temperature was 38.0 ℃. Atrial fibrillation with a rapid ventricular response (180/min) was observed after inpatient treatment was started. As the patient’s blood pressure reduced and respiratory status deteriorated, he was managed with artificial ventilation. His blood pressure could not be maintained without the use of vasopressor drugs, and intensive care was deemed to be required. Soon patient progressed to septic shock secondary to influenza and was transferred to the intensive care unit (ICU) for further management 53 h after the start of oral treatment for influenza. At the time of admission, during treatment with 0.1-μg/kg/min norepinephrine and 0.025-unit/min vasopressin, his blood pressure was 110/56 mmHg and his heart rate was 100 beats/min. Examination findings showed pain in the brachial muscle when grasping and pain from pressure on the trunk muscle, but no other abnormal findings were observed. The results of the blood test at the time of admission to the ICU are shown in Table 1.

**Table 1:** Laboratory data on admission Abbreviations: pH, power of hydrogen; PaO2, partial pressure of arterial oxygen; PaCO2, partial pressure of arterial carbon dioxide; HCO3, hydrogen carbonate; BE, base excess; WBC, white blood cell; Hb, hemoglobin; Ht, hematocrit; Plt, platelet; BUN, blood urea nitrogen; Cre, creatinine; CRP, c-reactive protein; CK, creatine kinase; IL-6; interleukin-6

			Biochemical analysis							
	Blood gas analysis				Reference range		Biochemistry			Reference range
		pH		7.306	7.380–7.460		BUN	46 mg/dL		8–20 mg/dL
		PaO_2_		498 mmHg	74.0–109.0 mmHg		Cre	2.15 mg/dL		0.65–1.07 mg/dL
		PaCO_2_		45.6 mmHg	32.0–46.0 mmHg		CRP	12.05 mg/dL		≤0.14 mg/dL
		HCO_3_		22.7 mEq/L	21.0–29.0 mEq/L		CK	48,137 U/L		4.9–6.0 U/L
		BE		-3.9mEq/L	-2–2 mEq/L		Myoglobin	99,850 ng/mL		<154.9 ng/mL
		Lactate		6.1 mmol/L	0.44–2.13 mmol/L		Troponin	0.019 ng/mL		<0.014 ng/mL
	Blood cell count						IL-6	46.7 pg/mL		< 4 pg/mL
		WBC		20,400/μL	3,300–8,600/μL		CK isozyme			
		Hb		18.3 g/dL	13.7–16.8 g/dL		MM	97 %		93–99 %
		Ht		53.2 %	40.7–50.1 %		MB	3 %		<6 %
		Plt		102,000/μL	158,000–348,000/μL		BB	0 %		<2 %
							Blood culture	Negative		
							Phlegm culture	Corynebacterium sp.		
							Urine culture	Negative		

**Figure 1:**
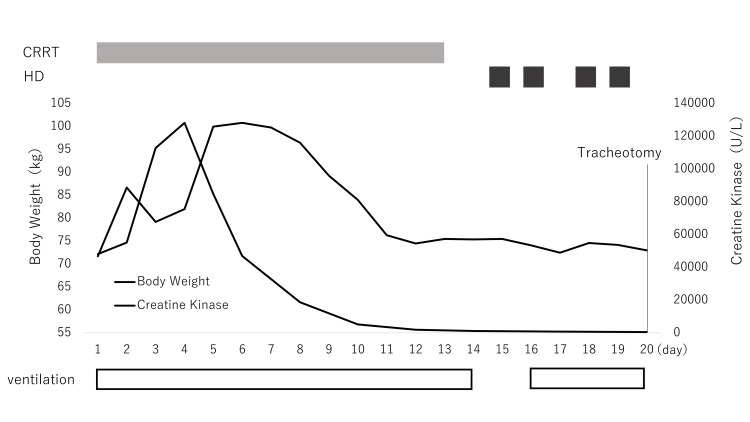
Clinical course Abbreviations: CRRT, continuous renal replacement therapy; HD, hemodialysis

With anuria continuing, the Kidney Disease Improving Global Outcomes criteria [3] were used to diagnose stage 3 acute kidney injury (AKI). It was accompanied by a notable increase in creatine kinase at 48,137 U/L. On the basis of clinical symptoms and the creatine kinase increase, the case was deemed to be a complication with rhabdomyolysis. Rhabdomyolysis and AKI were treated with fluid replacement using Ringer’s solution and continuous renal replacement therapy (CRRT). The treatment course for 20 days after ICU admission is shown in Figure 1.

The creatine kinase level continued to increase even after intensive treatment was started and increased by Day 4 after ICU admission. Large-dose fluid supplementation was required in respect of the hemoconcentration due to SCLS, and notable weight gain persisted for a week after ICU admission. The creatine kinase level gradually reduced beyond Day 4 after ICU admission. Vascular permeability slightly improved later. Furthermore, seven days after ICU admission, repeated tachycardiac paroxysmal atrial fibrillation occurred with the involvement of influenza myocarditis. Therefore, landiolol was administered, and rate control was performed. By this time, blood pressure had improved and norepinephrine was no longer needed. Early withdrawal from ventilator management was deemed to be difficult, and a tracheostomy was performed on Day 20 after ICU admission. Besides ICU-acquired weakness (ICUAW) [4], underlying muscle damage due to influenza infection-related rhabdomyolysis was suspected. Thus, after the tracheotomy, proactive rehabilitation was performed. With gradual improvement in respiratory muscle function, the patient could be weaned off from ventilator management on Day 31 after ICU admission. The patient was then transferred to the rehab hospital on Day 46 to continue rehabilitation. Furthermore, his renal function improved during the treatment course, and renal replacement therapy was not required from Day 27 after ICU admission. Paroxysmal atrial fibrillation during the acute phase stopped recurring.

## Discussion

SCLS accompanying influenza, as observed in our case, is rare, and few cases have been reported [5]. SCLS leads to anasarca, which was reported for the first time by Clarkson et al. [6] with a triad of signs, including concentrated blood due to the loss of plasma to tissue stroma, hypoalbuminemia, and hypotension. SCLS occurs in three phrases, the prodromal, acute extravasation, and recovery phases. In the acute phase, administration of pressor agents and abundant fluid replacement is required to prevent shock, but a certain level of oliguria is present [7]. During the recovery phase, vascular hyperpermeability improves, but pulmonary edema frequently occurs [8]. The clinical course of our case was typical for SCLS, except for AKI onset during the acute extravasation phase and insufficient urination. However, by appropriately removing water using CRRT, body fluid can be well managed. Of the deaths from SCLS, 75% occur during the acute phase, and myocardial edema and fatal arrhythmias are thought to be the main causes [9]. Influenza myocarditis may be the cause of arrhythmia [10]. Recurring tachycardiac atrial fibrillation during the acute phase indicates the possibility of influenza myocarditis. However, tachycardiac atrial fibrillation could be well managed with rate control using continuous intravenous infusion of landiolol. On the basis of our case, the following treatment strategies are considered necessary: 1. awareness of the possibility of influenza-triggered rhabdomyolysis or SCLS; 2. during the acute phase, use of abundant fluid supplementation and pressor agents for circulation management to promote vascular permeability and prevent arrhythmia; 3. during the recovery phase, because the intravascular volume rapidly increases. If the kidney is damaged and sufficient diuresis cannot be achieved, fluid control should be attained via renal replacement therapy.

## Conclusions

Influenza is an acute viral disease that affects individuals of all ages worldwide. Its clinical symptoms are related to all organs, beyond those affected by the typical respiratory syndrome. Extrapulmonary complications, such as SCLS and rhabdomyolysis, as in our case, greatly affect patient prognosis. Therefore, knowledge of the pathophysiology and clinical course of influenza infection complicated by rhabdomyolysis or SCLS is important in the treatment during the acute phase.
